# The German Crown Prince

**Published:** 1887-11-19

**Authors:** 


The Hospital

A Weekly Institutional Journal of

Science, Medicine, and Philanthropy.

*For Hospitals, Asylums, Medical Practitioners, Students, Nurses, Families, Parishes, Congregations, and the Charitable Public.*

Vol. III. No. 60.] [November 19, 1887.

### The German Crown Prince.

Exalted rank may lift a man above sordid cares, but cannot save him from those mortal dangers to which all mankind are alike exposed. The meanest person in a hospital who is the subject of painful and progressive disease, commands the sympathy of all who see or know of his condition. In like manner, when one of those terrible maladies which know no respect of persons seizes upon a prince of the blood, the whole world looks on with anxious eyes and saddened and fearful heart. What can any man do in the grasp of such a deadly enemy as has now laid its hand upon the Crown Prince? He can but resign himself with submission to such saving measures as the medical science and skill of his time afford. Happily that science and skill can cope with the disease in such a way that if cure be within the range of human power, cure will be effected; but if otherwise, there will be no irrational barbarities to endure, no ignorant charlatanism to drive the patient to despair.

It can hardly be expected, human nature being such as it is, and the difficulties of the case being what they are, that all the physicians, including as they do also pathologists and laryngologists, will quite coincide as to the treatment to be pursued and the periods when each step shall be taken. But what the world has a right to expect, and even to insist upon, is that every one of those who may be called to take part in the conduct of the case shall rid himself entirely of professional jealousies and national prejudices. Such displays of human weakness and infirmity may be natural; but in the presence of sickness and suffering which may prove mortal they cannot be called noble—they are, in truth, miserable and base. The medical science and skill of Europe are now playing their part on a stage which is visible to the whole world. If the selected physicians play their part worthily, putting personal and local considerations out of sight, and devoting themselves with singleness of purpose to the great end of saving the illustrious patient's life, they will win for themselves the world's gratitude and honourable regard. But if any of them at such a crisis in the history of a great nation and its prince should be so poor in magnanimity as to display the faintest wish to make personal advantage his ruling motive, or the detraction of a brother an end to be desired, he will not only deserve but secure the stern contempt of all right-minded men. In this world men must and will fight and contend against one another; but in the presence of the conflict between a dread disease and its victim all lesser conflicts should be hushed. It is to be hoped for the sake of human nature, as well as for the honour of a great profession, that whatever differences of opinion may be entertained and expressed, differences of sentiment there will be none.

The Crown Prince's illness is a grief to the civilised world; to the Princess and her family it is terrible. As a wife and mother, the Princess has made her name famous among the world's famous women. In no rank has greater devotion been witnessed to those duties which make home the centre of all that is most gentle and beautiful in human life. It will surprise no one that she is her husband's most devoted attendant, and that she gives up to him not only health and strength, but the sleep which those who watch and wait most sorely need. This Royal family have now lived long before the world, and from the earliest period of their married life until now have steadily advanced in the general esteem. In their own Germany esteem has grown to affection, and thousands of families among great and small, are waiting with anxious apprehension the further development of the disease. Humanly speaking, it is difficult to imagine any life on the prolongation of which more momentous interests seem to depend than on that of the Crown Prince. The destined head of one of the most powerful States the world has ever known—a State surrounded and environed by every variety of doubtfully-friendly or semi-hostile elements; he himself the representative of a policy of watchful defence, but also of peaceful national development, if that may be possible; a man whose judgment is always inspired by intelligence and controlled by experience and conscience—he of all men, under such circumstances, it would seem the world can least spare. Yet it is he of all men upon whom, it is to be feared, has seized one of the most deadly maladies with which mankind have to contend. What is the use of multiplying words at such a time? Surely it is less than nothing and vanity, except that the Crown Prince himself and his family and country cannot but be moved to deep gratitude by the spectacle to which the newspapers all point, of whole nations look on in anxious sympathy, fearing the worst, yet striving to hope for the best. The nations, and the individuals of which they are composed, have something to learn from the brave and cheerful fortitude of the husband, and also from the pathetic devotion of the wife and mother. "One touch of nature makes the whole world kin." Never did the familiar words of Shakespeare find fitter illustration than at this moment and in the household of kings. No height of greatness can make any human being more than a man; no depth of degradation can make a sane man less. Sorrow, pain, mortal disease, attack all alike, and unite all together in a common bond of grief and submission to the unyielding hand of destiny.

Science and medical skill, it may be taken for granted, will do what is possible to eradicate the disease, and in the worst event to allay suffering and give skilled and patient aid. But how soon the resources of the physician and the scientist reach their utmost limits! What does science mean at this great and conspicuous crisis? It means the best skill and judgment of one poor mortal man, or at most of half-a-dozen such. What can it accomplish in this impressive case, when called upon for the sublimest effort it can make? Perhaps nothing! Or possibly at most the prolongation of life for a few brief months or years. Is there nothing then for the Prince to look forward to but the powerlessness of science and the hopeless termination of a great and distinguished career? The Prince puts his trust in God. It is a striking spectacle for thoughtful, and even for thoughtless men. He does not despair, or resolve to end the fight with destiny by self-destruction. He puts his trust in God. Who knows but that in the secret counsels of the Highest this very event may be made to accomplish more in the intellectual and spiritual history of mankind than a long reign of uneventful peace or a brilliant period of outwardly glorious but destructive and desolating war!

Men, wrapped up in the interests of the day, and shut in by the environing cloud of their own personal concerns, do not see the ordered march of the great world's mind or the goal to which High Providence may be leading them. But that there are "many things in heaven and earth not dreamt of in our philosophy," and that there is a "divinity that shapes our ends, rough hew them how we will"—these are the convictions common to all men—these the profound instincts which stay the mind in the midst of the most tremendous assaults of time and fate. That these convictions give fortitude to the Prince's mind, and hope and consolation to him and to all who have claims upon his affections, is surely a source of the profoundest satisfaction to all who have hearts to feel or minds to look forward to the dread unknown. Great as is the crisis which seems to be impending in the fate of the Prince and of his country, it is not a gloom without any ray of light. That faith which, in common with the lowliest of his countrymen, he feels to be based on everlasting truth and right, will make him victorious in that dread conflict in which both prince and peasant alike must sooner or later engage.

